# Assessing the Impact of Salt Reduction Initiatives on the Chronic Disease Burden of Singapore

**DOI:** 10.3390/nu13041171

**Published:** 2021-04-01

**Authors:** Ken Wei Tan, Sharon Esi Duoduwa Quaye, Joel Ruihan Koo, Jue Tao Lim, Alex R. Cook, Borame L. Dickens

**Affiliations:** Saw Swee Hock School of Public Health, National University of Singapore, Singapore 117549, Singapore; ephtkw@nus.edu.sg (K.W.T.); ephsedq@nus.edu.sg (S.E.D.Q.); ephkoor@nus.edu.sg (J.R.K.); ephljt@nus.edu.sg (J.T.L.)

**Keywords:** obesity, intervention modelling, acute myocardial infarction, stroke, salt intake

## Abstract

Globally, many countries are facing an increasing burden of chronic disease due to ageing populations, of which cardiovascular disease forms a large proportion. Excess dietary sodium contributes to cardiovascular disease risk and requires intervention at a population level. This study aimed to quantify the impact of several salt reduction initiatives on population health over a 30-year horizon using GeoDEMOS, a population model from Singapore. Four interventions were modelled in four demographic groups in 2020 for a total of 16 intervention scenarios. The effect of 0.5, 2.0, and 4.0 g/day reductions in daily salt consumption, along with adherence to the World Health Organization guidelines of a maximum of 5.0 g of salt each day, was modelled in the entire population, including the overweight and obese, the elderly, and diabetics. In each scenario, the number of averted incident cases of acute myocardial infarction and stroke, along with the disability-adjusted life years up to 2050, was monitored. We found 4.0 g/day reductions in salt consumption were the most effective when implemented across the entire population, resulting in 24,000 averted incident cases of cardiovascular disease and 215,000 disability-adjusted life years over 30 years. This is a large figure when compared with the 29,200 projected annual incident cases of cardiovascular disease in 2050. When targeted at specific high-risk demographic groups, the largest effects were observed in the overweight and obese, with the same intervention yielding 10,500 averted incident cases of cardiovascular disease and 91,500 disability-adjusted life years. Quantifying the benefits of salt reduction initiatives revealed a significant impact when administered across the entire population or the overweight and obese. Health promotion efforts directed toward sustainably reducing salt consumption will help to lower the chronic disease burden on the healthcare system in years to come.

## 1. Introduction

High salt intake increases blood pressure and hypertension risk [1,2], acting as precursors to cardiovascular and renal diseases [3,4,5], which are known as major global causes of mortality and disability. Although the World Health Organization (WHO) recommends that adults consume 5 g or less of salt per day, people’s daily salt intake exceeds this recommendation in 181 out of 187 countries [6]. Singapore is no exception, with a mean daily intake of 8–9 g reported in 2018 [7,8], where cardiovascular diseases contributed 14.2% of the island nation’s total disability-adjusted life years (DALYs), which is the topmost ranking among all diseases [9]. The proportions of diabetic sufferers and those classified as obese are also steadily growing [10,11,12,13], where increased chronic disease risk exists among these subpopulations. In 2017, 8.6%, 8.7%, and 21.5% of Singaporean adults aged 18 to 69 years were diabetic, obese, and hypertensive, respectively [9].

Given Singapore’s rapidly ageing population and the proportion of diabetic and obese individuals, a large proportion of the population is already at a high risk of developing cardiovascular diseases and stroke, leading to an even greater disease burden for the city state [14,15,16]. By 2050, the elderly population (aged 65 years and above) is expected to constitute approximately half of Singapore’s population [17], and one in six adults aged 18 to 65 will be diabetic, translating to approximately 1 million people [18]. The prevalence of obesity is additionally forecasted to rise to 15.9% of the population by 2050 [18].

Policy makers within Singapore have responded by rolling out several programs and campaigns to reduce chronic disease risk by enforcing the benefits of salt intake reduction [19,20,21]. In 2011, Singapore’s Health Promotion Board (HPB) launched a War on Salt campaign by partnering with local food manufacturers to develop a healthier salt with 25% less sodium than regular salt, among other initiatives, under the FINEST FOOD programme. HPB has since introduced the Healthier Dining Programme for food and beverage companies to increase community awareness of the risks in high salt consumption, and the Healthier Choice Symbol Programme for packaged foods with the Lower in Sodium label on foods containing 25% lower sodium levels in comparison with similar products [22]. As of yet, however, no estimates have been calculated to quantify the impact of large-scale salt-reduction interventions on the chronic disease outcome of the Singaporean population.

In this study, we aimed to model and compare the impacts of four interventions of varying amounts of salt reduction on acute myocardial infarction (AMI) and stroke incidence for the whole population and three high risk groups separately: the elderly defined as persons aged 65 and above, overweight individuals defined as persons with a body mass index (BMI) greater than 23, and diabetic individuals. We examined the impacts in terms of the number of incident cases averted and the reduction in the disease burden, and modelled the future incidence of AMI and stroke under the interventions until 2050.

## 2. Materials and Methods 

We modelled the effects of salt intake reduction across 16 different scenarios, with 4 different population groups as the target populations and 4 different levels of reduction. For the population model, we used GeoDEMOS, which projects the Singapore population and their diabetic status until 2050 [18,23]. The agent-based model contains a synthetic population where individual demographic attributes and lifetime risk in disease inheritance can be monitored. It operates on a yearly time step and is able to project to 2050, accounting for fertility and mortality rates based on census information available from the Singapore Department of Statistics [24]. Data were obtained from the Singapore Myocardial Infarction Registry and the Singapore Stroke Registry to create disease models for AMI and stroke with outcomes stratified by age, sex, ethnicity and diabetic status recorded (full details in Supplementary Figures S1 and S2).

### 2.1. Modelling of Salt Intake

We used information from the National Nutrition Survey 2010, conducted by the health promotion board [25], to obtain information on salt consumption. The survey provides estimated salt intake in g/day based on sodium levels from urinary excretion. The stratum-specific distributions of salt intake for each permutation of age group (18–29, 30–39, 40–49, 50–59 and 60–69 years), sex and ethnicity (Chinese, Malay and Indian) were then estimated. Salt intake for individuals aged 70 years and above were assumed to match the oldest age group of 60–69 years and likewise for those below 18 years of age. Distributions of salt intake by demographic strata can be found in Supplementary Table S1.

### 2.2. Modelling the Effect of Salt Restriction

A salt restriction policy aims to modify behavior and induce a quantifiable change in salt intake, which impacts chronic disease risk. The salt reduction relative risk values were adapted from a Canadian study [26], where a large reduction in daily salt consumption of 4.0 g/day led to a relative risk reduction of 4% for AMI and 10% for stroke. These relative risk reductions were scaled proportionally to the individual reduction in daily salt consumption and the effects assumed to have a uniform effect on all demographic groups in Singapore. In tandem with the existing risk models of AMI and stroke, we were able to model the effects of various salt restriction policies on the risk that individuals developed AMI and stroke to forecast different scenarios.

### 2.3. Interventions

In Singapore, no large-scale estimates for interventions and their impact on salt consumption exist; therefore, possible scenarios were inferred from literature. A systematic review by Hyseni et al. demonstrated that multi-component campaigns, including dietary counselling, reformulation to reduce salt content, health promotion, and nutritional labelling, are expected to reduce salt consumption by 2.0 g/day on average [27]. This estimated reduction is further supported by two meta-analyses by Brunner et al. and Hooper et al. which reported similar values of 1.8 g/day after 18 months and 2.0 g/day after 60 months, respectively [28,29]. Numerous public health interventions and improvements in technology in Japan resulted in a reduction of 3.9 g/day from 14.5 g/day in 1972 to 10.6 g/day in 2010 over a 40 year horizon, supporting a reduction of 4.0 g/day as a suitable upper bound [30].

A separate systematic review by Trieu et al. suggested that 0.5 g/day was the 25th percentile in reported reductions in salt intake out of 12 countries over varying timescales, which was used here as a conservative estimate of the effectiveness of salt reduction initiatives [31]. Another intervention we investigated was a hypothetical complete adherence to the World Health Organization recommended amount of 5.0 g/day [32]. We additionally modelled these three reductions in salt consumption (0.5, 2.0, and 4.0 g/day) as conservative, expected, and optimistic scenarios, respectively, as well as the adherence scenario in different strata of the population.

The baseline was assumed to be a uniform adoption of the initiatives for each individual in the population. We also simulated scenarios where the salt reduction initiatives targeted high-risk strata of the population, such as the overweight (defined as having a body mass index > 23.0 [33]), the elderly aged 65 years and above, and diabetics. These populations are well-established as being at greater risk of having AMI and stroke. All salt reduction interventions were modelled to have occurred in 2020. The four salt reduction scenarios with the four high-risk strata brought the total number of intervention scenarios to sixteen (described in Table 1).

### 2.4. Outcome Measures

In each of the sixteen scenarios, we monitored the annual number of averted incident cases of AMI and stroke compared with the baseline incidence. Additionally, we calculated the cumulative averted cases of AMI and stroke to quantify the effects of the interventions over 30 years. To measure the reduction in disease burden, we computed the number of disability-adjusted life years (DALYs) in each of the scenarios until 2050. The method we used to calculate DALYs is described in detail in Supplementary Information 1.

## 3. Results

**Figure 1:**
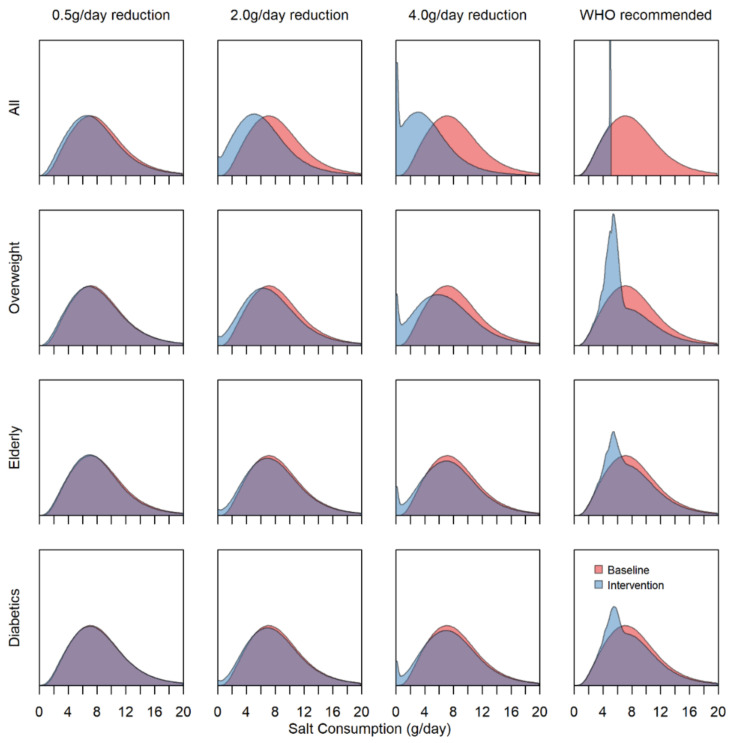
Population-level salt intake density plots at baseline and under each of the 16 intervention scenarios. The area under the curve indicates the proportion of the population with a given salt consumption in g/day.

The distribution of salt consumption in the population at the baseline and after the various interventions differs substantially in their efficacy (Figure 1 and Figure S3). Regardless of demographic strata, a 0.5 g/day reduction in salt consumption has a negligible effect on the population, as opposed to the 2.0 and 4.0 g/day reductions, which have visible effects on population salt consumption. At reductions of 2.0 and 4.0 g/day, when restricted to specific high-risk strata, we observed larger decreases to the distribution of ~1.0 and 2.0 g on average for the overweight strata compared with negligible decreases of <0.5 g in the elderly and diabetics. For the WHO recommended intake group of interventions, complete adherence in the entire population will have the largest impact on the salt consumption distribution. Nevertheless, even when targeted at the high-risk strata, there are clear changes in the population salt consumption, with the most prominent change to the distribution appearing in the overweight strata.

**Figure 2:**
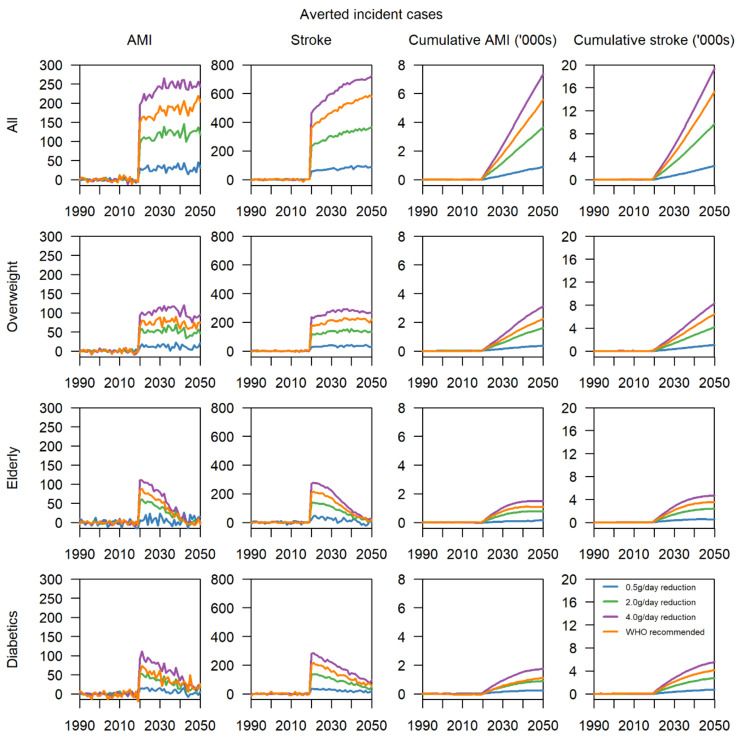
Annual averted cases and cumulative cases of acute myocardial infarction (AMI) and stroke from 1990 to 2050 for the 16 intervention scenarios from 1990 to 2050.

When applied across the entire population, the most effective intervention was found to be a 4.0 g/day reduction with around 200 averted cases of AMI per year, and 600 averted cases of stroke per year, where the increasing trend is a result of the ageing population of Singapore (Figure 2). Over 30 years, 6500 AMI and 17,500 stroke cases would be averted. Less extreme interventions of 0.5 and 2.0 g/day showed similar dose–response curves, resulting in 1000 and 3500 averted AMI cases, as well as 2500 and 9500 averted stroke cases, respectively. Quantitatively, adherence to the WHO recommended intake is slightly less effective than the 4.0 g/day reduction intervention, with 5500 averted cases of AMI and 15,500 averted cases of stroke cumulatively (Figures S4 and S5). When targeting overweight individuals in 2020, a reduction of 4.0 g/day likewise yields the greatest benefits, with 100 and 250 averted cases of AMI and stroke per year, and 3000 and 7500 cumulatively, respectively. Likewise, for this bundle of scenarios, a dose–response relationship is present. Scenarios involving elderly and diabetics in 2020 display diminishing returns, with the greatest decrease in annual incident cases observed in the year of implementation. At a 4.0 g/day reduction, there were approximately 100 and 250 averted cases of AMI and stroke, respectively, in 2020, equivalent to being implemented in the overweight individuals. By 2050, in the elderly, both values decrease to almost 0, whereas in diabetics we observed that there are still around 100 averted cases of stroke per year. In 2020, approximately one-third of AMI and stroke cases were either elderly or diabetics, but the proportion is projected to decline steadily towards 2050 as elderly and diabetics already have higher than average mortality and are likely to pass away of other causes within the 30 years from 2020 to 2050.

**Figure 3:**
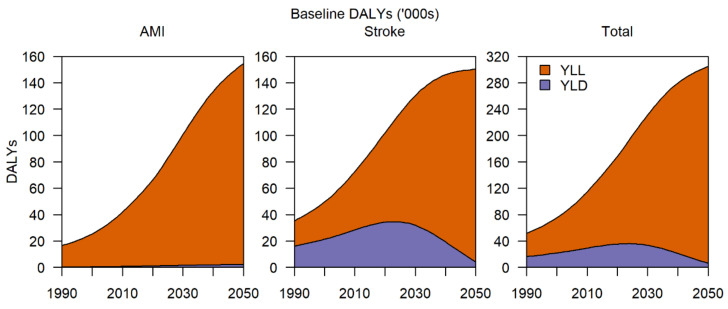
Baseline disability-adjusted life years (DALYs) for AMI and stroke per year, broken down into years of life lost (YLL) and years lost to disability (YLD) from 1990 to 2050.

In terms of disease burden, presently without any intervention, AMI and stroke contribute 160,000 DALYs, which is expected to increase to 310,000 DALYs by 2050 (Figure 3). For AMI, the bulk of the DALYs comes from years of life lost (YLL) as assuming affected individuals survive after treatment, there are no major decreases to quality of life, as opposed to stroke where, even after initial treatment, there are long-term impacts on quality of life. For stroke, the breakdown between YLL and years lost to disability (YLD) is about equal, although by 2050, the average age of developing stroke would have increased to the point where YLD contributes much less to DALYs compared to the YLL as a result of increased mortality and a larger number of elderly in the population.

**Figure 4:**
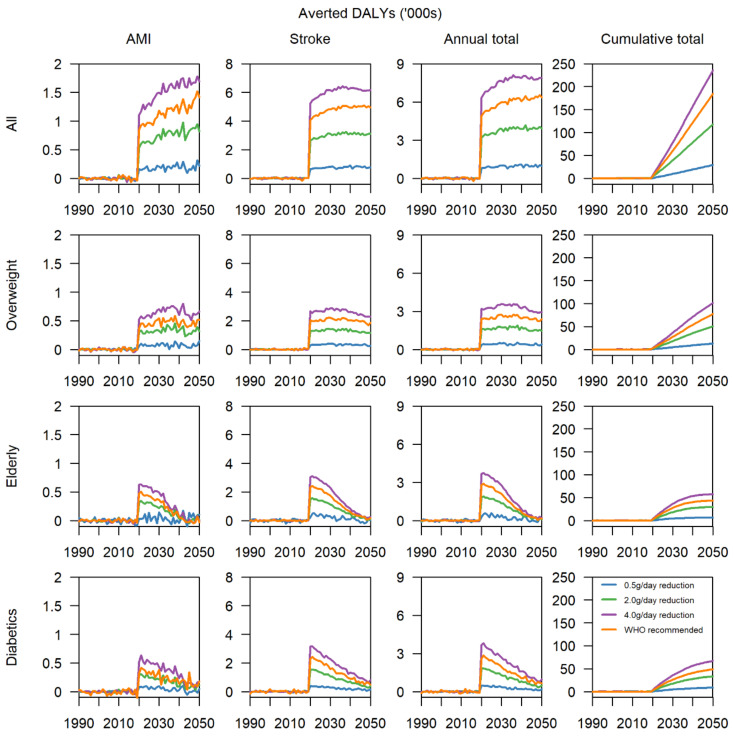
Total averted DALYs for AMI, stroke, and both chronic conditions combined per year and cumulatively.

The trends in averted DALYs show that if a reduction of 4.0 g/day is achieved in the entire population, in 30 years, the cumulative number of averted DALYs would be almost 200,000, following a continuing upward trend (Figure 4 and Figure S6), with about 1500 DALYs from AMI and 6000 DALYs from stroke per year. Even at a moderate reduction of 2.0 g/day, the cumulative number of averted DALYS is expected to surpass 100,000, with 1000 annually averted DALYs from AMI and 3000 from stroke. For cause-specific DALYs, AMI contributes 20% and stroke 80% of the total disease burden annually. When the intervention is applied to overweight individuals, despite being applied to a fraction of the population in 2020, there are a cumulative total of 100,000 averted DALYs by 2050 if a 4.0 g/day reduction is implemented, an effect size similar to 2.0 g/day in the entire population. Overweight individuals adhering to the WHO recommended intake also yield a cumulative total of 80,000 DALYs and may be more feasible for implementation. In the elderly and diabetics, the cumulative total of averted DALYs tapers off at 50,000 or less regardless of intensity of the intervention due to death from other causes, evidenced by the sharp initial spike in annually averted DALYs in 2020, then declining year on year. Reductions in salt consumption of 4.0 g/day in both high-risk groups are forecasted to avert a total of about 60,000 DALYs in both high-risk groups, 25% of the total effect size when implemented in the entire population and 60% when implemented in the overweight strata.

## 4. Discussion

We assessed the effects of four daily salt reduction interventions of 0.5 g, 2.0 g, 4.0 g, and 5.0 g on population groups for AMI and stroke. The four population groups explored to determine the relative effectiveness of population-wide policies were the entire Singapore population, the elderly (defined as persons aged 65 years and above), overweight individuals (defined as persons with a body mass index (BMI) greater than 23.0), and diabetic individuals. From all these intervention options, the implementation of a 4.0 g reduction in daily salt intake population-wide averted the greatest incidence and DALYS for stroke and AMI. Should a population-wide intervention be unfeasible, however, a 4.0 g reduction in the overweight would be highly effective.

The region of Southeast Asia generally has some of the world’s lowest prevalence of obesity [34], but with many countries experiencing rapid economic growth and urbanization, these rates are beginning to trend upward. Singapore’s closest neighbours, Indonesia and Malaysia, and several others in the region, such as Vietnam, have seen a major increase in their rates of obesity and the proportion of those overweight [35,36]. Obesity is typically viewed as a disease of the developed world, since it usually accompanies rising incomes and urbanization [37]. Although this is still largely true, the rates of obesity are steadily increasing in many developing countries, particularly in cities and areas that are experiencing rapid economic progress [38]. An increase in the availability of and accessibility to processed foods—particularly foods categorized as empty calories—and a more sedentary lifestyle are characteristics of the changing socioeconomic landscape contributing to the burgeoning weight problem [37,38]. Although the largest sources of dietary sodium in Southeast Asian nations are from traditional salted and fermented foods and from added salts [39,40], processed foods with their high sodium content [41] also contribute to dietary sodium. Several studies have revealed that a high salt intake is an independent risk factor of obesity or overweight [42,43] and is also strongly and independently associated with an increased risk of cardiovascular diseases and congestive heart failure among the overweight [44,45]. With overweight and obesity being independent risk factors for cardiovascular diseases [10,13], there is much benefit to be derived from designing salt reduction interventions aimed at this high-risk group.

Almost 1 in 10 Singapore adults aged 18 to 65 years are obese and around 3 in 10 are considered to be overweight [9]. As of 2017, overweight and obesity accounted for 6.4% of the nation’s total DALYs, rising from 3.9% in 1990 [9]. Overweight individuals account for a substantial proportion of the population at risk, with a cumulative total of 100,000 averted DALYs by 2050. This is comparable to the impact observed when a 2.0 g reduction per day is applied to the entire population. As this group is at the highest risk for cardiovascular disease, which is the largest contributor to Singapore’s disease burden [9], substantial DALYs can be averted. For this subgroup, our results estimate that 18,000 DALYs can be averted for AMI and 73,500 for stroke (Figure 3). Lower reductions of 0.5 g, however, have a relatively negligible impact, with 2500 and 10,500 DALYs, respectively, averted, supporting higher reduction interventions. Moderate reductions of 2.0 g could be considered as an initial intervention as 10,000 AMI and 39,000 stroke DALYs can be averted with plans for larger scale reductions in the near future.

Intervention scenarios involving the elderly did not yield great benefits in averting cases of AMI and stroke over the 30-year period from 2020 to 2050, since individuals in this high-risk stratum may have had long-term high salt intake and consequent organ damage. Regardless, this group should be prioritised as a key target group for salt-reduction interventions through careful management of their daily salt intake, which can effectively reduce their risk of mortality as well as improve their quality of life [46,47].

Given that most nations have an average daily salt intake higher than the daily recommended amount, many have devised and implemented national salt reduction strategies, but only few have made any significant progress toward reducing salt consumption. Many barriers exist, including the lack of motivation, unawareness of current recommendations, and an underestimation of consumption [27,48]. Japan, the U.K., and Finland are among the few that have achieved substantial reductions through national programmes. Japan experienced a significant reduction in hypertensive rates and stroke mortality beginning in the 1960s, which were attributed to a successful reduction in dietary salt intake [3,49]. These reductions in hypertension and stroke mortality have been ongoing, although paradoxically, the average salt intake has remained largely unchanged since the 1980s with no further reductions [50,51]. The U.K. saw a 15% reduction in salt consumption over seven years after adopting several strategies [52]. Finland’s salt reduction efforts saw a 40% reduction in salt consumption from 1970 to 2008, as well as a decrease in hypertensive rates and 80% reduction in deaths from stroke [53]. The strategies employed by these countries include product reformulation, nutritional labelling, and information campaigns.

Current salt reduction initiatives in Singapore are similar, observing, in particular, an increasing popularity of the healthier choice food products [54]. Singapore’s population works long hours at an average of 44.7 h per week [55]; frequently eat at food courts called hawker centres, where they are unable to control the sodium input; and frequently buy processed and ready-to-eat foods in super markets [56]. New salt intake initiatives for Singapore will need to be tailored for its resident population. In contrast to most western countries, where the greatest proportion at ~75% of dietary sodium comes from manufactured and restaurant-prepared foods, for Asian countries, the cooking process and sauces are the highest contributors [39,40,50,57]. In 2011, an estimated 60% of the Singaporean population’s salt intake was from table salt and sauces, whereas processed foods contributed only 37% of dietary salt or sodium intake [8]. Whereas HPB has focused on partnering with a number of these food and beverage providers to provide healthier options through its Healthier Dining Programme [58], more radical policies may have to be implemented to reduce a sizeable proportion of the population’s sodium intake by at least 2.0 g.

This study does have some limitations. The relative risk reductions were assumed to have affected all strata of the population equally, whereas, in reality, a dose–response relationship would be observed. Individuals with a much higher baseline salt consumption would benefit more from salt reduction interventions than those who are only slightly overconsuming salt in their diets. However, in the absence of studies that accurately report this relationship, the crude effect had to be used. This modelling study also assumed complete adherence to the intervention to obtain the reported outcome. Pragmatically, a significant proportion of the population would remain unreached, depending on the various implementation strategies, thereby diminishing the effect. Another limitation is how long the risk reductions take to occur. Intuitively, a sudden change in lifestyle will not yield immediate returns, but instead improve over time, although the model assumes that the relative risk reductions are applied instantaneously within a single year, regardless of an individual’s past consumption.

Despite these limitations, our results clearly support the prioritisation of the overweight and obese populations as key target intervention groups of salt-reduction programmes in Singapore, for both an effective and potentially cost-effective solution. Additionally, while targeting high-risk populations to modify their salt consumption patterns, consideration should be given to how public health promotion can be reworked to also focus on building healthy and sustainable salt consumption habits within the younger population. This could be achieved through a comprehensive and large-scale public health education, a regulation of salt or sodium content in processed foods and encouraging the use of healthier ingredients by the food and beverage industry particularly in hawker centres (Singapore’s local food centres), food courts, and fast food restaurants. Policy measures taken have largely been moving in this direction with the various programmes that HPB has already introduced. However, these efforts need to be revaluated and ramped up given that the average daily salt intake has stayed about the same since the launch of the War on Salt campaign in 2011. Overall, as approximately 9 in 10 Singaporeans continue to consume almost twice the recommended daily intake of salt [7], the introduction of such evidence-based policies discussed in this study are required to continue lowering salt consumption, which we find should be primarily targeted at high-risk populations, such as the overweight.

## Figures and Tables

**Table 1:** Overview of the 16 intervention scenarios. WHO, World Health Organization; BMI, body mass index.

Intervention 1: Daily Salt Reduction of 0.5 g	Intervention 2: Daily Salt Reduction of 2.0 g	Intervention 3: Daily Salt Reduction of 4.0 g	Intervention 4: WHO Recommended Daily Salt Intake of 5.0 g or Less
All individuals aged 18 and above reduced daily salt consumption by 0.5 g	All individuals aged 18 and above reduced daily salt consumption by 2.0 g	All individuals aged 18 and above reduced daily salt consumption by 4.0 g	All individuals aged 18 and above adhere to the WHO recommended daily salt intake of 5.0 g or less
Elderly aged 65 and above reduced daily salt consumption by 0.5 g	Elderly aged 65 and above reduced daily salt consumption by 2.0 g	Elderly aged 65 and above reduced daily salt consumption by 4.0 g	Elderly aged 65 and above adhere to the WHO recommended daily salt intake of 5.0 g or less
Overweight individuals with BMI > 23.0 reduced daily salt consumption by 0.5 g	Overweight individuals with BMI > 23.0 reduced daily salt consumption by 2.0 g	Overweight individuals with BMI > 23.0 reduced daily salt consumption by 4.0 g	Overweight individuals with BMI > 23.0 adhere to the WHO recommended daily salt intake of 5.0 g or less
Diabetic individuals reduced daily salt consumption by 0.5 g	Diabetic individuals reduced daily salt consumption by 2.0 g	Diabetic individuals reduced daily salt consumption by 4.0 g	Diabetic individuals adhere to the WHO recommended daily salt intake of 5.0 g or less

## Data Availability

Data on the National Nutrition Survey 2010 can be requested from the Health Promotion Board, Singapore at HPB_Mailbox@hpb.gov.sg. Demographic data is available upon request.

## Supplementary Materials

The following are available online at https://www.mdpi.com/article/10.3390/nu13041171/s1. Table S1: Salt consumption in g/day estimated from the National Nutrition Survey 2010, Supplementary Information 1: Calculation of DALYs, Figure S1: Projected incidence of AMI from 1990 to 2050, Figure S2: Projected incidence of stroke from 1990 to 2050, Figure S3: Salt consumption in mg/day in demographic strata, Figure S4: Cumulative averted AMI incident cases, Figure S5: Cumulative averted stroke incident cases, Figure S6: Cumulative averted DALYS.
