# Supplementary material for: Assessing the Impact of Salt Reduction Initiatives on the Chronic Disease Burden of Singapore
Source: Nutrients. 2021 Apr 1;13(4):1171. doi: 10.3390/nu13041171 (PMC8065991; doi:10.3390/nu13041171)
Supplement: Supplementary file 1 [file nutrients-13-01171-s001.zip › nutrients-1152225-supplementary.pdf]

Supplementary Table 1

AGE GROUP	CHINESE MALES	CHINESE FEMALES	MALAY MALES	MALAY FEMALES	INDIAN MALES	INDIAN FEMALES
**18 – 29**	9.11 (0.21)	9.03 (0.39)	8.90 (0.42)	8.73 (0.52)	9.07 (0.51)	8.56 (0.50)
**30 – 39**	9.08 (0.30)	9.06 (0.40)	9.03 (0.36)	8.58 (0.51)	8.77 (0.39)	8.30 (0.59)
**40 – 49**	9.17 (0.37)	8.74 (0.45)	8.97 (0.42)	8.76 (0.40)	8.73 (0.42)	8.50 (0.47)
**50 – 59**	8.98 (0.29)	8.74 (0.46)	8.93 (0.34)	8.43 (0.54)	8.63 (0.54)	8.50 (0.54)
**60 – 69**	8.98 (0.32)	8.83 (0.55)	8.81 (0.64)	8.46 (0.57)	8.55 (0.60)	8.24 (0.44)

Salt consumption in g/day was estimated from urinary sodium measurements in the National Nutrition Survey 2010. The table contains the means and standard deviations (in brackets) of estimated salt consumption in mg/day which were drawn from a lognormal distribution based on data from the National Nutrition Survey 2010. Parameters are stratified by age group, ethnicity and gender. In the projections, other ethnic groups used the parameters from the Chinese ethnicity due the Chinese being the majority of the population.

Supplementary Information 1

Disability-adjusted life years (DALYs) were calculated using the standard formula of computing years of life lost and years lost to disability with 3% future discounting. For the calculation of years of life lost, life expectancy by gender from 1990 to 2018 was obtained from the Department of Statistics, Singapore and is publicly available. The life expectancy for years after 2018 was assumed to be that of 2018. For individuals that developed acute myocardial infarction (AMI) or stroke, the years of life lost was computed using the difference between their simulated age at death and the life expectancy for that year. If an individual was older than the life expectancy at the year of death, the years of life lost was zero.

The years lived with disability for AMI was modelled to be 3 months following onset of an event with a disability weight of 0.395, following the methodology of the Singapore Burden of Disease 2010. Stroke was assumed to have moderate permanent impairments and surviving individuals were assigned a disability weight of 0.630 for each year, also following the Singapore Burden of Disease 2010[3].

Supplementary Figure S1

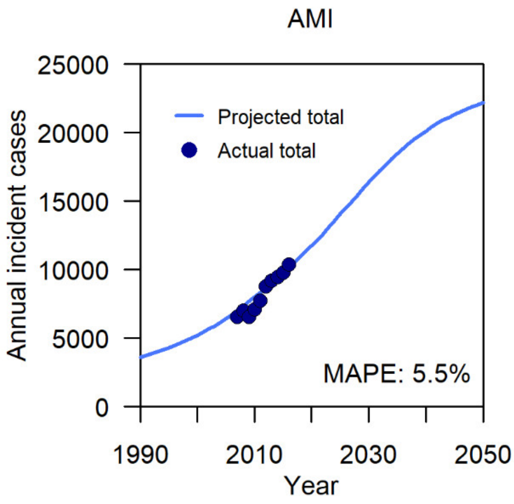

Projected incidence of AMI from 1990 to 2050 at baseline using an agent-based population model against the National Registry of Diseases Office (NRDO) reported incident cases from 2007 to 2016[1]. Mean absolute percentage error (MAPE) of the model was 5.5% across the 10 years of data.

Supplementary Figure S2

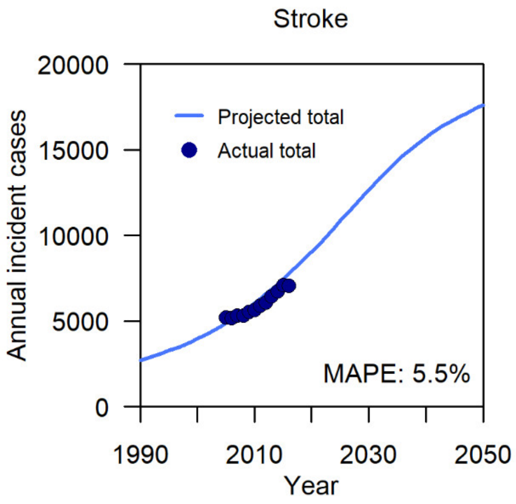

Projected incidence of stroke from 1990 to 2050 at baseline using an agent-based population model against the National Registry of Diseases Office (NRDO) reported incident cases from 2005 to 2016[2]. Mean absolute percentage error (MAPE) of the model was 5.5% across the 12 years of data.

Supplementary Figure S3

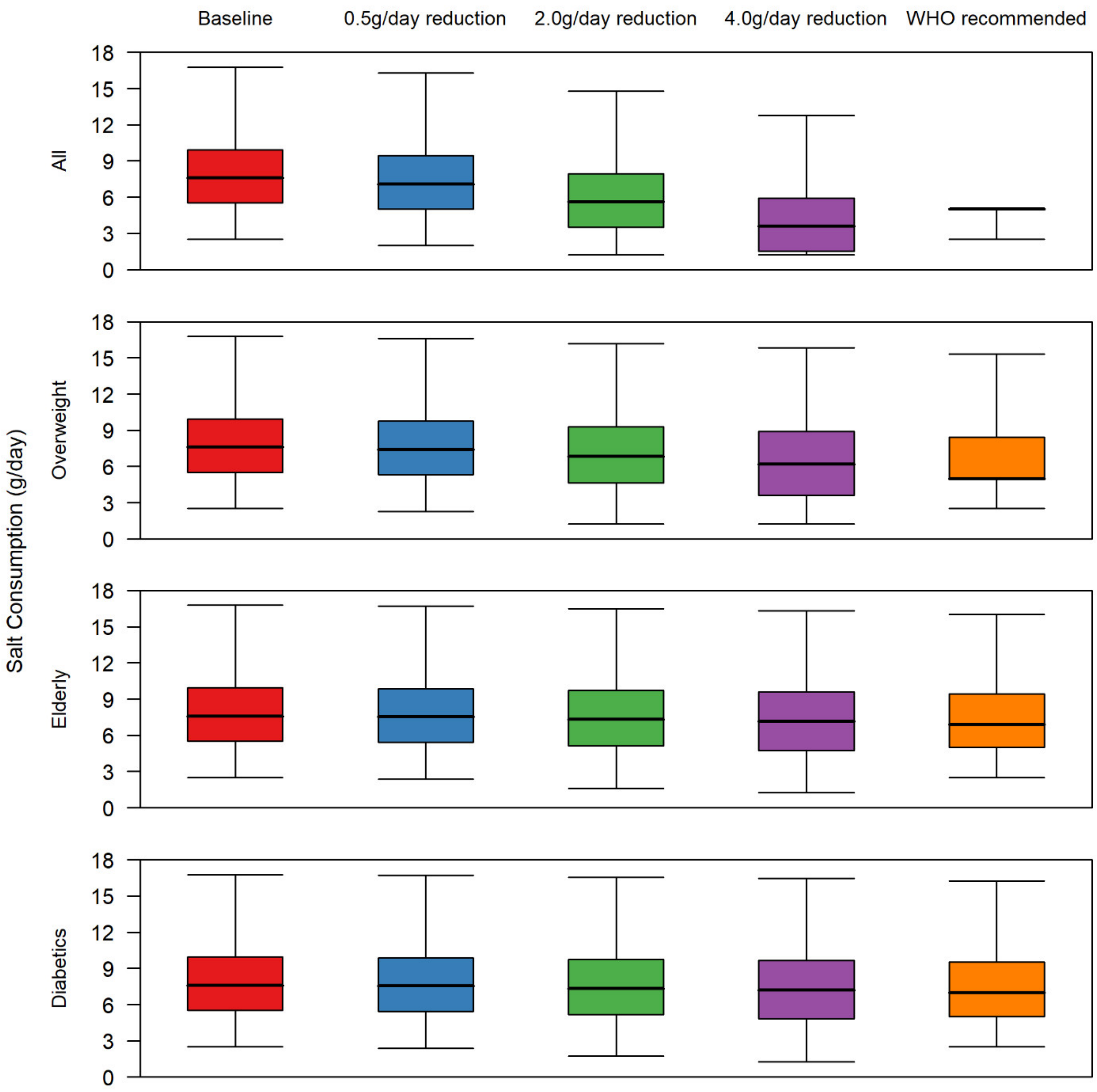

Boxplots of population salt consumption at baseline and under each of the 16 intervention scenarios. The five metrics represented in each boxplot are the 2.5, 25, 50, 75 and 97.5 percentiles of salt consumption, to indicate the distribution of salt consumption in the population.

Supplementary Figure S4

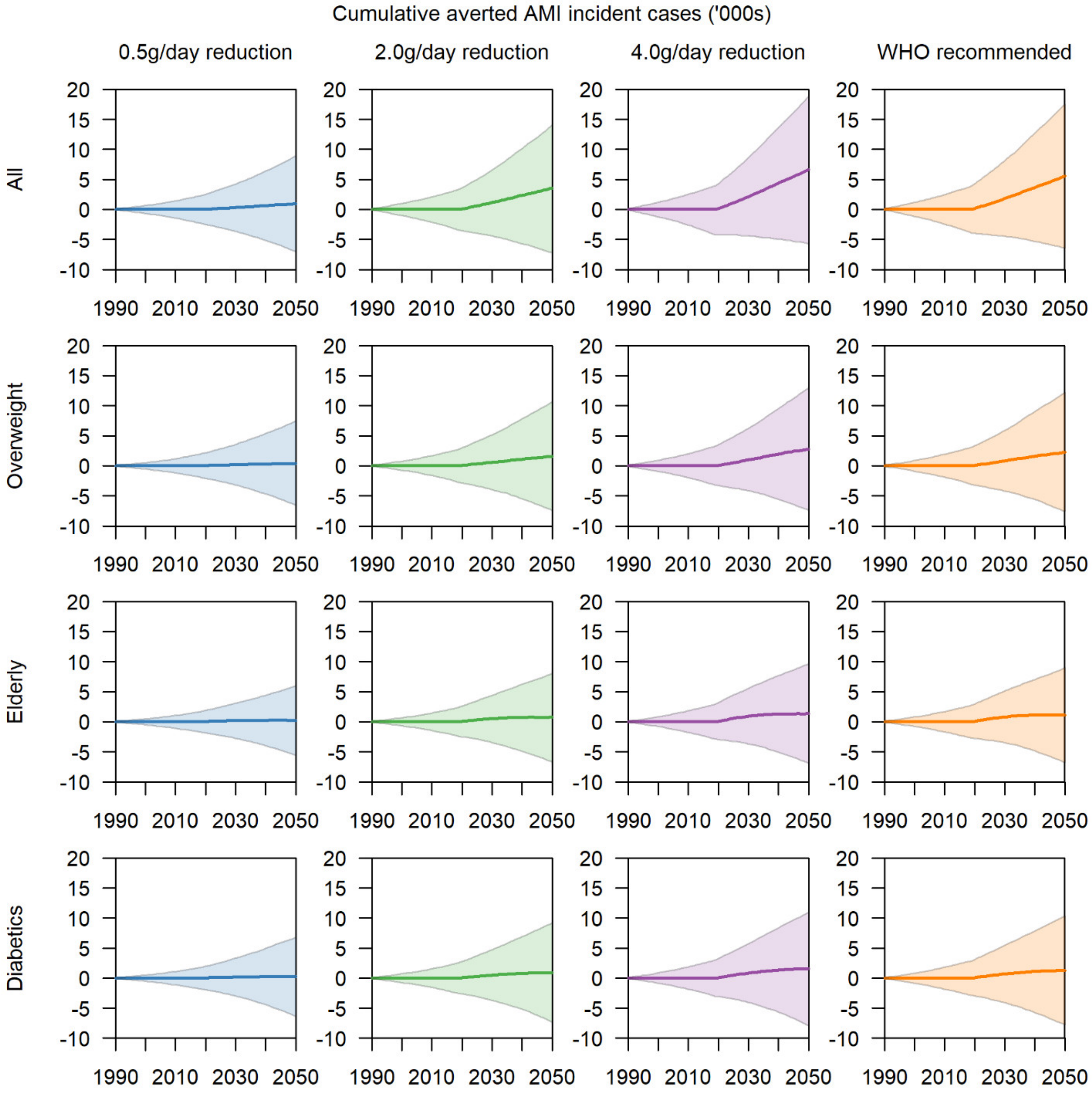

Cumulative averted AMI cases in thousands from 1990 to 2050 under the 16 intervention scenarios.

Supplementary Figure S5

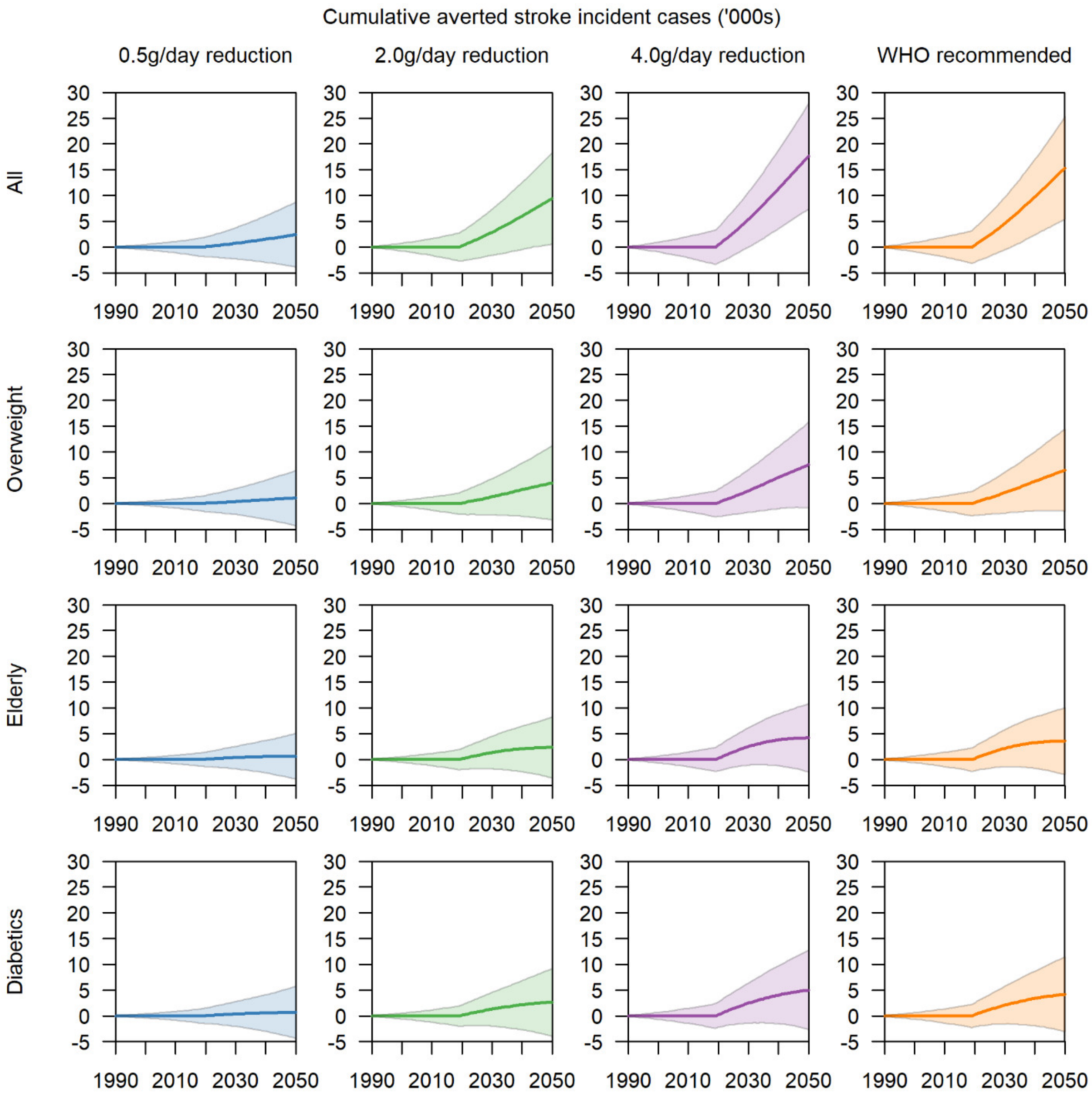

Cumulative averted stroke cases in thousands from 1990 to 2050 under the 16 intervention scenarios.

Supplementary Figure S6

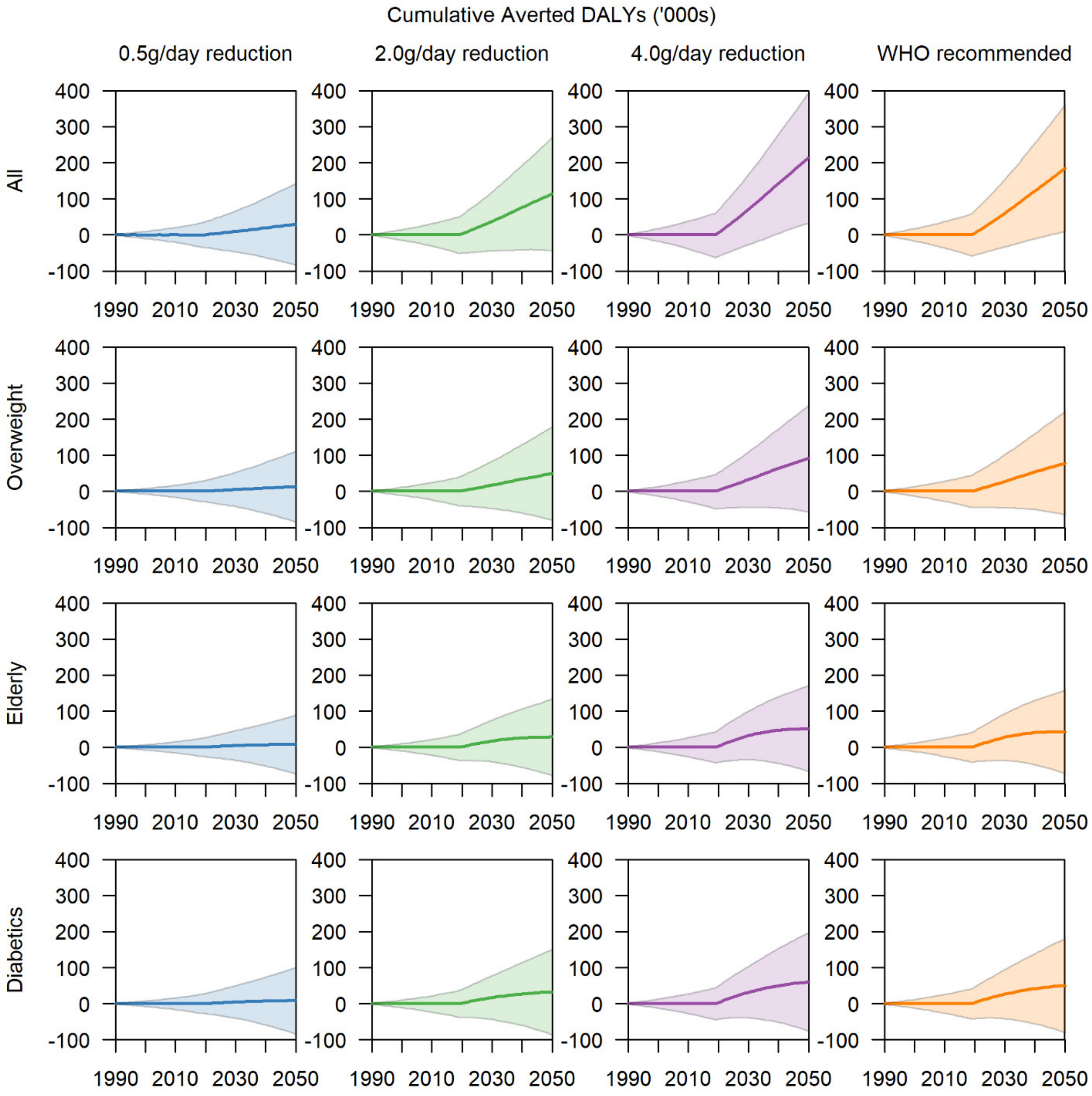

Cumulative averted DALYs in thousands from 1990 to 2050 under the 16 intervention scenarios.

References

1. Ministry of Health, Singapore. Singapore Myocardial Infarction Registry Annual Report 2017. (2019).
2. Ministry of Health, Singapore. Singapore Stroke Registry Annual Report 2017. (2019).
3. Singapore Burden of Disease and Injury Study Working Group, Singapore, Ministry of Health & Epidemiology and Disease Control Division. *Singapore burden of disease study 2010.* (2014).
